# Loss of Canonical *Smad4* Signaling Promotes *KRAS* Driven Malignant Transformation of Human Pancreatic Duct Epithelial Cells and Metastasis 

**DOI:** 10.1371/journal.pone.0084366

**Published:** 2013-12-27

**Authors:** Lisa Leung, Nikolina Radulovich, Chang-Qi Zhu, Dennis Wang, Christine To, Emin Ibrahimov, Ming-Sound Tsao

**Affiliations:** 1 Department of Medical Biophysics, University of Toronto, Toronto, Ontario, Canada; 2 Department of Laboratory Medicine and Pathobiology, University of Toronto, Toronto, Ontario, Canada; 3 Ontario Cancer Institute/Princess Margaret Hospital, and University Health Network, University of Toronto, Toronto, Ontario, Canada; Northwestern University, United States of America

## Abstract

Pancreatic ductal adenocarcinoma (PDAC) is the fourth most common cause of cancer death in North America. Activating *KRAS* mutations and *Smad4* loss occur in approximately 90% and 55% of PDAC, respectively. While their roles in the early stages of PDAC development have been confirmed in genetically modified mouse models, their roles in the multistep malignant transformation of human pancreatic duct cells have not been directly demonstrated. Here, we report that *Smad4* represents a barrier in *KRAS*-mediated malignant transformation of the near normal immortalized human pancreatic duct epithelial (HPDE) cell line model. Marked *Smad4* downregulation by shRNA in *KRAS* ^G12V^ expressing HPDE cells failed to cause tumorigenic transformation. However, KRAS-mediated malignant transformation occurred in a new HPDE-TGF-β resistant (TβR) cell line that completely lacks Smad4 protein expression and is resistant to the mito-inhibitory activity of TGF-β. This transformation resulted in tumor formation and development of metastatic phenotype when the cells were implanted orthotopically into the mouse pancreas. *Smad4* restoration re-established TGF-β sensitivity, markedly increased tumor latency by promoting apoptosis, and decreased metastatic potential. These results directly establish the critical combination of the *KRAS* oncogene and complete *Smad4* inactivation in the multi-stage malignant transformation and metastatic progression of normal human HPDE cells.

## Introduction

Pancreatic cancer is the fourth leading cause of cancer death in North America with an overall five year survival rate of <5% [1]. Pancreatic tumors primarily arise from the duct and are referred to as pancreatic ductal adenocarcinoma (PDAC). The progression from normal duct epithelium to invasive carcinoma is characterized by the accumulation of genetic changes which advance precursor lesions called pancreatic intraepithelial neoplasias (PanINs) [2]. *KRAS* mutations are found in >90% of invasive PDAC and during the multi-stage PDAC carcinogenesis, its occurrence has been shown to precede the inactivation of tumor suppressors *p16* (95%), *p53* (75%), and *Smad4* (55%) [3]. Active *KRAS* stimulates downstream pathways involved in cell survival, motility, and proliferation [4]. Genetically modified mouse models (GEMMs) engineered to express the *KRAS* ^G12D^ oncogene in the developing pancreas can recapitulate the advancement of PanIN lesions seen in patients, however only a subset of mice develop invasive and metastatic PDAC [5,6]. The incomplete progression to invasive adenocarcinoma indicates that *KRAS* alone is insufficient for malignant transformation of the pancreatic duct epithelium.

The TGF-β signaling pathway is frequently disrupted in pancreatic cancer, and *Smad4* loss is found in ~55% of PDAC has been associated with advanced disease and distant metastases [7,8]. *Smad4* plays a crucial role in the canonical TGF-β signaling pathway. Briefly, the TGF-β ligand binds to its receptor complex resulting in the phosphorylation of Smad2 and Smad3 which enables their binding to Smad4. This Smad oligomer forms part of the transcriptional complex that regulates processes such as cell cycle progression and extracellular matrix protein expression [9]. Targeted *Smad4* inactivation in the mouse pancreas does not initiate tumorigenesis, however concomitant *Smad4* loss and *KRAS* ^G12D^ expression leads to the rapid development of PanIN lesions and cystic tumors [10-12]. 

We utilized cells derived from normal human pancreatic duct to dynamically study the contribution of these genetic changes in pancreatic carcinogenesis *in vitro*. Previously we reported that *KRAS* ^G12V^ expression in an immortalized near normal human pancreatic duct epithelial (HPDE) cell line led to stochastic and incomplete tumorigenic transformation [13]. The H6c7 cell line was a clone of the HPDE6-E6E7 cell line that was immortalized by retroviral transduction of the HPV16-E6E7 genes, which deregulated G1-S checkpoint and *p53* pathways [14]. In the current study, we have investigated the consequences of *Smad4* loss alone and in combination with *KRAS* ^G12V^ oncogene to further delineate its role in the context of multi-stage human pancreatic duct cell carcinogenesis and malignant progression. To examine *Smad4* loss we utilized shRNA targeted against *Smad4* in the H6c7 cell line, and established a novel cell line derived from the H6c7 cell line called H6c7-TβR (abbreviated as TβR), which completely lacks *Smad4* protein expression. 

## Materials and Methods

*Cell culture and* in vitro *assays*. The H6c7 cell lines were derived from normal human pancreatic duct explant and immortalized using amphotropic retrovirus, LXSN16E6E7, containing the E6 and E7 genes of HPV-16 [14]. All H6c7 derived cell lines were grown in keratinocyte serum-free media (Lonza, Basel, Switzerland) as previously described [15]. TGF-β sensitivity was assessed after TGF-β exposure (R&D Systems, Minneapolis, MN, USA), as previously described [13]. Invasion assays were performed as previously described [15]. Cells were treated with 26 μM 5-azacytidine or vehicle (50% acetic acid; Sigma Aldrich, Oakville, ON, Canada) for 7 days prior to RNA or protein isolation. 

### Smad4 small hairpin RNA gene silencing

Smad4 expression was stably downregulated by shRNA retroviral transduction method using Phoenix-amphotropic packaging cell line (ATCC, Manassas, VA, USA). The shRNA sequences were ligated into the pSUPER GFP retrovirus vector after linearization with Bgl*II* and Hind*III* (New England Biolabs, 

Whitby, ON, Canada). The shRNA oligonucleotides used were: S4KD1: ggacaatatgtctattacgaa; S4KD2: gcagtgactttgtatagagaa; S4KD3: actgctaaattctatgttaaa; S4KD4: ggtggagagagtgaaacattt; and non-silencing (NS) control siRNA sequence: ttctccgaacgtgtcacgt (Qiagen, Venlo, Netherlands ). 

### KRAS and Smad4 expression constructs

KRAS^G12V^ expression was performed as described before [16]. Smad4 expression construct was purchased from Open Biosystems (Ottawa, ON, Canada) and the plko.Smad4-EGFP vector was generated using our modified Gateway cloning system (Invitrogen, Burlington, ON, Canada) [17].

### PCR. Quantitative real-time RT-PCR (qPCR):

Total RNA was isolated from cells and PCR was performed as described before [15]. *For Smad4 gene copy number*: genomic DNA (gDNA) was isolated from cells using the DNAeasy kit (Qiagen, Toronto, Ontario). Gene copy number was determined as described before [18]. Briefly, Smad4 copy number was assessed by comparing the C_T_ values from three primer sets against the standard curve. gDNA isolated from the H6c7 cell line served as the control. Copy number was calculated using Stratagene Mx3000P (Agilent, Mississauga, Ontario). *Methylation specific PCR (MSP*): MSP was perfomed using gDNA isolated from cells and bisulfite treated gDNA using the EpiTect bisulfite kit (Qiagen). Bisulfite treated gDNA was amplified using using AmpliTaq (Applied Biosystems, Burlington, ON, Canada) and primers were designed using the MethPrimer program [19]. Primer sequences are listed in Table S1 in File S1.

### Animals

All studies were conducted using protocols approved by the Animal Care Committee of the Ontario Cancer Institute. Tumor growth and implantation was assessed as described before [13]. Briefly, 2x10^6^ cells were suspended in 50 µl medium supplemented with 10% or 20% Matrigel for subcutaneous or pancreatic orthotopic implantation in NOD SCID mice, respectively (BD, Mississauga, ON, Canada). Mice were euthanized once subcutaneous tumors reached a length of 1.5 cm, or if mice presented with deteriorating clinical condition (abdomen distension, weight loss exceeding 20% of normal body weight, and hunched appearance). 

### Immunoblotting

Immunoblotting was performed as previously described [15]. Briefly, proteins were applied to SDS polyacrylamide gels and assayed for KRAS activity (Upstate, Burlington, ON, Canada), KRAS, PAI-1 Smad4 (Santa Cruz Biotechnology, Dallas, TX, USA); phospho- and total Smad2 and Smad3 (R&D Systems); GAPDH (Cell Signaling); β-actin (Sigma Aldrich). Visualization was accomplished by using horseradish peroxidase-linked anti-rabbit and anti-mouse secondary antibodies (Cell Signaling, Boston, MA, USA) and ECL-Prime Western blotting kit (GE Biosciences, Pittsburgh, PA, USA). 

### Immunohistochemistry

Immunohistochemistry was performed as previously described [15]. AE1/AE3 human cytokeratin 7 and cytokeratin 20 antibody (Dako, Burlington, ON, Canada), Smad4 (Santa Cruz Biotechnology), cleaved caspase-3 (Cell Signaling), MIB1/Ki67 (Dako), human chromosome 17 SISH detection kit (Ventana, Tuscon, AZ, USA), and cleaved PARP (Abcam, Cambridge, MA, USA) were used as directed in product protocols. AE1/AE3 and/or chromosome 17 SISH positively stained sections were used to count metastases and Aperio ImageScope (Vista, CA, USA) software was used to determine the area of the metastases. The positive pixel algorithm in the ImageScope program was utilized to quantify the degree of positive staining. 

### Microarray analysis

Transcriptional profiling was performed on the H6c7, TβR, TβR-pBp, TβR-KRAS, TβR-KRAS-EV, and TβR-KRAS-Smad4 cell lines using the Illumina HumanOmni5-Quad, respectively (Illumina, San Diego, CA, USA). The microarray data were normalized using log_2_-transformation and quantile normalization. Moderated paired t-tests were used to compare samples and controls. ASCAT was used to identify copy number amplification and deletion regions. Common differences in fold changes that were > 2-fold were included in our analyses carried out using SAS v9.2. GO and KEGG annotations were carried out using the Database for Annotation, Visualization and Integrated Discovery (*DAVID*) v6.7.

### Statistical analysis

Tumor and cell growth were analyzed using linear regression, survival was calculated using Cox-proportional hazard tests, Fisher’s exact test was utilized to compare rates of metastasis between TβR-KRAS-EV and TβR-KRAS-Smad4 orthotopic models, differential immunostaining between the TβR-KRAS-EV and TβR-KRAS-Smad4 orthotopic xenografts were analyzed using student t-test, and data as indicated were analyzed using ANOVA, and student t-test using GraphPad Prism 5 (La Jolla, CA, USA). Data in figures are presented as the means ± SEM. P values <0.05 were considered significant. 

## Results

### Incomplete Smad4 knockdown by shRNA and KRAS^G12V^ expression promotes invasion, but not tumorigenicity.

**Figure 1:**
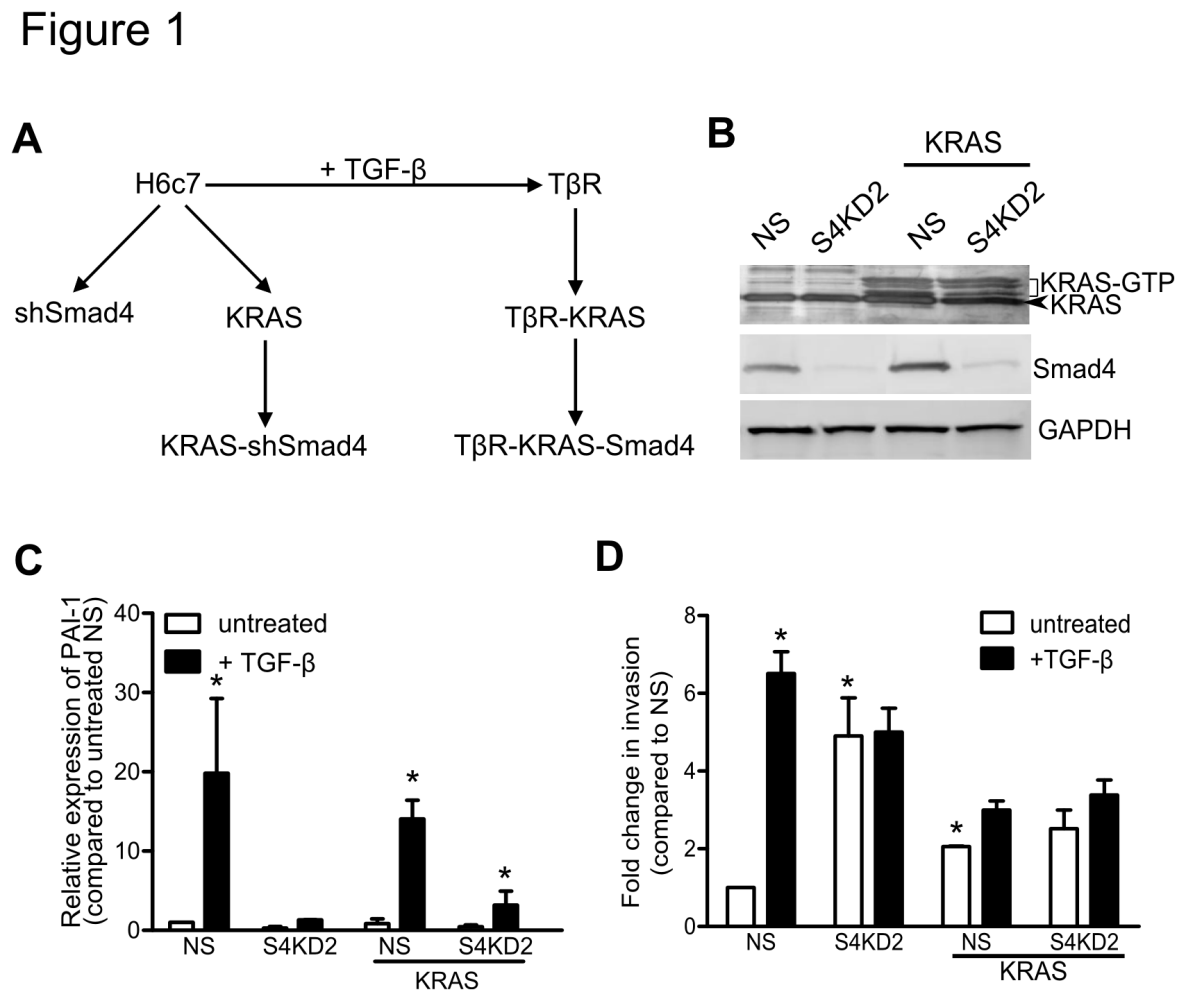
Smad4 knockdown and KRAS^G12V^ expression in the H6c7 cell line. (**A**) A schema of introducing KRAS^G12V^ and Smad4 loss using the H6c7 cell line to produce the shSmad4, H6c7 KRAS shSmad4, TβR, TβR-KRAS , and TβR-KRAS -Smad4 cell lines. (**B**) Immunoblot of RAS-GTP, KRAS, and Smad4. GAPDH was used as a loading control. (**C**) PAI-1 mRNA expression after 48 hours of TGF-β stimulation (n=3). (**D**) Invasion assays through Matrigel coated membranes incubated with and without TGF-β (n=6). (* denotes significant differences between the test and control NS samples, and between treatment and no treatment groups two-way ANOVA and Bonferroni’s post hoc tests or paired student t-test; p<0.05; data are presented as mean ± SEM) .

To assess the consequences of *Smad4* deficiency in the H6c7 cells, we stably transduced four different retroviral short hairpin RNA (shRNA) *Smad4* constructs (S4KD) and a non-specific (NS) shRNA construct (Figure 1A and Figures S1A-B). *Smad4* expression was significantly attenuated by the shRNA sequence, S4KD2 (Figure 1B). To determine if *Smad4* inactivation synergises with *KRAS* oncogene activation, *Smad4* was knocked down by 80% in a *KRAS* ^G12V^ expressing H6c7 cell line (H6c7-KRAS; Figures 1A-B and Figures S1C-E). *KRAS* was demonstrated to be active, and mRNA expression of *Smad2, Smad3, TGFBR1, and TGFBR2* remained unchanged after *KRAS* ^G12V^ expression and/or *Smad4* knockdown (Figures 1B and Figure S1F). Importantly, TGF-β-induced *PAI-1* and *Smad7* mRNA expression was diminished in H6c7-S4KD2 and H6c7-KRAS-S4KD2 cells (Figure 1C and Figure S1G). Regardless of *KRAS* expression, knocking-down *Smad4* abrogated TGF-β sensitivity, but did not affect cellular proliferation (Figures S1H-I). *Smad4* downregulation or *KRAS* ^G12V^ expression enhanced invasion through Matrigel coated Boyden chambers (Figure 1D). TGF-β induced invasion in the parental H6c7 cells (NS) cells, but had no effect on cells after *Smad4* and/or *KRAS* expression were modified. Despite reduced *Smad4* expression (>80%), the H6c7-S4KD2 and H6c7-KRAS-S4KD2 cells failed to form tumors in non-obese diabetic (NOD) Severe combined immune deficient (SCID) mice (Table 1). 

**Table 1:** The effect of KRAS and Smad4 expression on the invasiveness, TGF-β sensitivity, and tumorigenicity of the TβR cell lines.

				Subcutaneous				Orthotopic			
	In vitro									Metastases	
Cell Line	Invasion (compared to H6c7)	TGF-β sensitive	No. animals	Tumor weight (g)	Mean Survival (d)	No. animals	Tumor weight (g)	Mean Survival (d)	Liver	Spleen	Kidney
H6c7	-	Yes	0/5	-	-	-	-	-	-	-	-
H6c7-S4KD2	↑	No	0/5	-	-	-	-	-	-	-	-
H6c7-S4KD2-KRAS	↑	No	0/5	-	-	-	-	-	-	-	-
H6c7-KRAS	↑	Yes	0/5	-	-	-	-	-	-	-	-
H6c7-TβR	↑	No	0/5	-	-	-	-	-	-	-	-
H6c7-TβR-Smad4	↓	Yes	0/5								
H6c7-TβR-pBp	↑	No	0/5	-	-	0/5	-	-	-	-	-
H6c7-TβR-KRAS	↑	No	20/20	0.92 ± 0.04	-	13/13	1.21 ± 0.03	36	2 (15)	10 (77)	-
H6c7-TβR-pBp-Smad4	↓	Yes	0/5	-	-						
H6c7-TβR-KRAS EV	↑	No	10/10	0.75 ± 0.06*	27.5	16/16	1.29 ± 0.14*	33*	5 (31)	16 (100)*	7 (44)*
H6c7-TβR-KRAS Smad4	↑	Yes	9/10	0.39 ± 0.08	73	19/19	0.91 ± 0.09	48	2 (10)	7 (37)	2 (10)

The phenotypic differences between the cell lines, and the incidence of tumor and metastasis formation was assessed in NOD SCID mice after subcutaneous or orthotopic implantation of two million cells. [*-*, indicates not assessed; * denotes significant differences between the test (Smad4) and control (EV) samples, significance was assessed by paired t-test or Fisher’s exact test, p<0.05, data are presented as mean ± SEM; S4KD, Smad4 shRNA knockdown; pBp, p-BABE-puro (control vector); EV, empty vector; d, day; g, gram; and no., number.]

### Establishment of a TGF-β resistant H6c7 cell line

**Figure 2:**
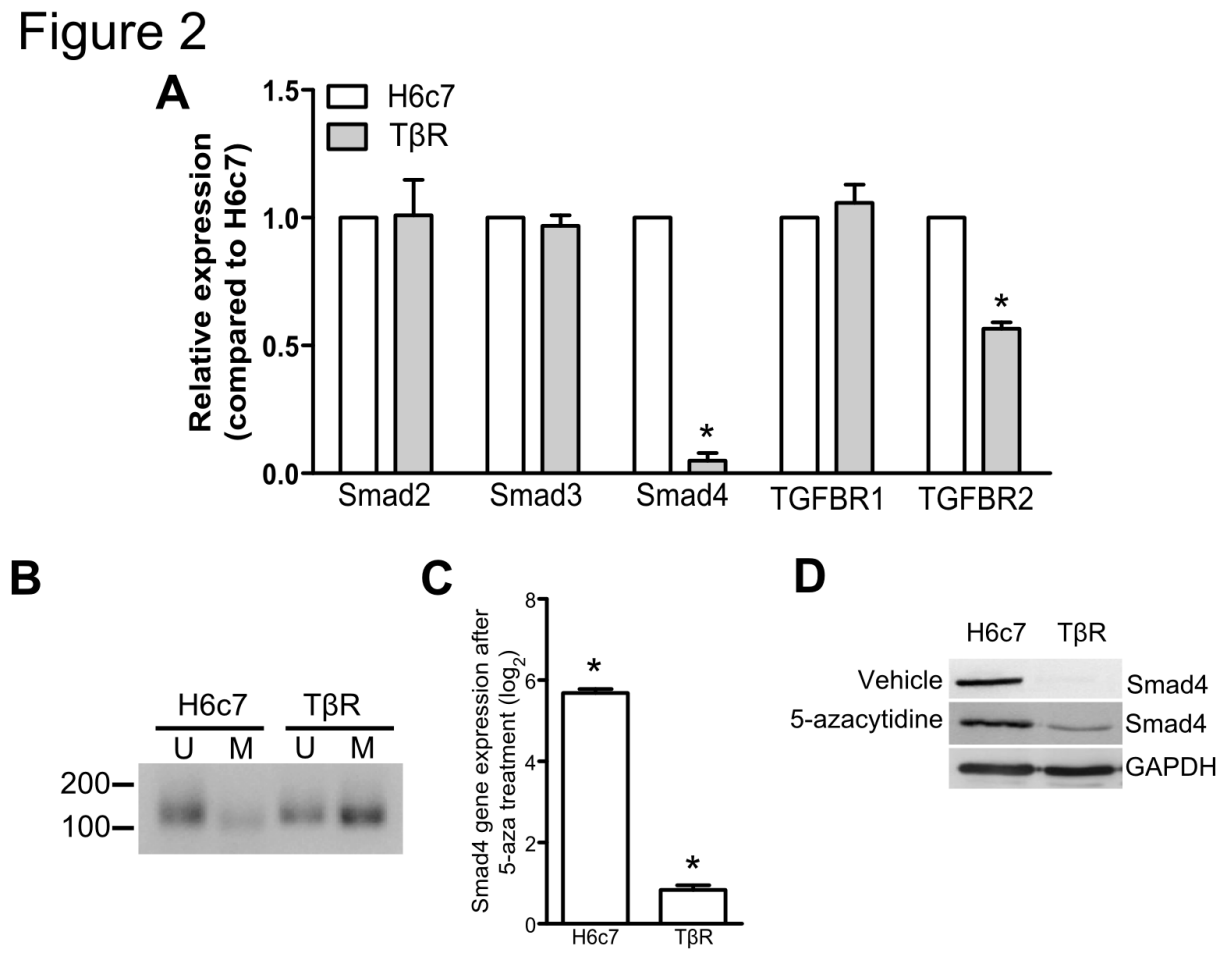
Characterization of the TβR cell *line*. (**A**) Smad and TGF-β receptors expression were assessed by qPCR and compared to the control H6c7 cell line. (**B**) Methylation specific PCR was performed on bisulfite treated gDNA isolated from H6c7 and TβR cells. Where U and M represent unmethylated and methylated, respectively. H6c7 and TβR cells were treated with 5-azacytidine and Smad4 expression was assessed by (**C**) qPCR and (**D**) immunoblotting. (* denotes significant differences between H6c7 and TβR cell lines or treated compared to vehicle where appropriate, student t-tests, p<0.05, n=3; data are presented as mean ± SEM) .

Since our above findings revealed that incomplete *Smad4* expression loss does not permit *KRAS*-mediated transformation of H6c7 cells, we then developed a novel cell line that completely lacks Smad4 expression. This was achieved by culturing H6c7 cells in medium with incremental concentrations of TGF-β until resistance to growth inhibition was attained, thus this cell line was named H6c7-TGF-β-Resistant (TβR; Figure 1A). Compared to H6c7 cells, qPCR revealed undetectable *Smad4* mRNA expression in the TβR cell line and a 30% reduction in Smad4 copy number (Figure 2A). We investigated other possible mechanisms affecting *Smad4* expression loss since the loss of expression was only partially accounted for by copy number loss. Methylation specific PCR (MSP) performed on bisulfite treated genomic DNA isolated from the H6c7 and TβR lines demonstrated promoter methylation in the TβR line, and treatment with methyltransferase inhibitor, 5-azacytidine, partly restored *Smad4* expression (Figures 2B-D). Altogether these results suggest that continuous culture of H6c7 cells in TGF-β led Smad4 silencing through gene copy loss and promoter methylation.

### KRAS^G12V^ expression in the H6c7-TβR cell line induces tumorigenicity

**Figure 3:**
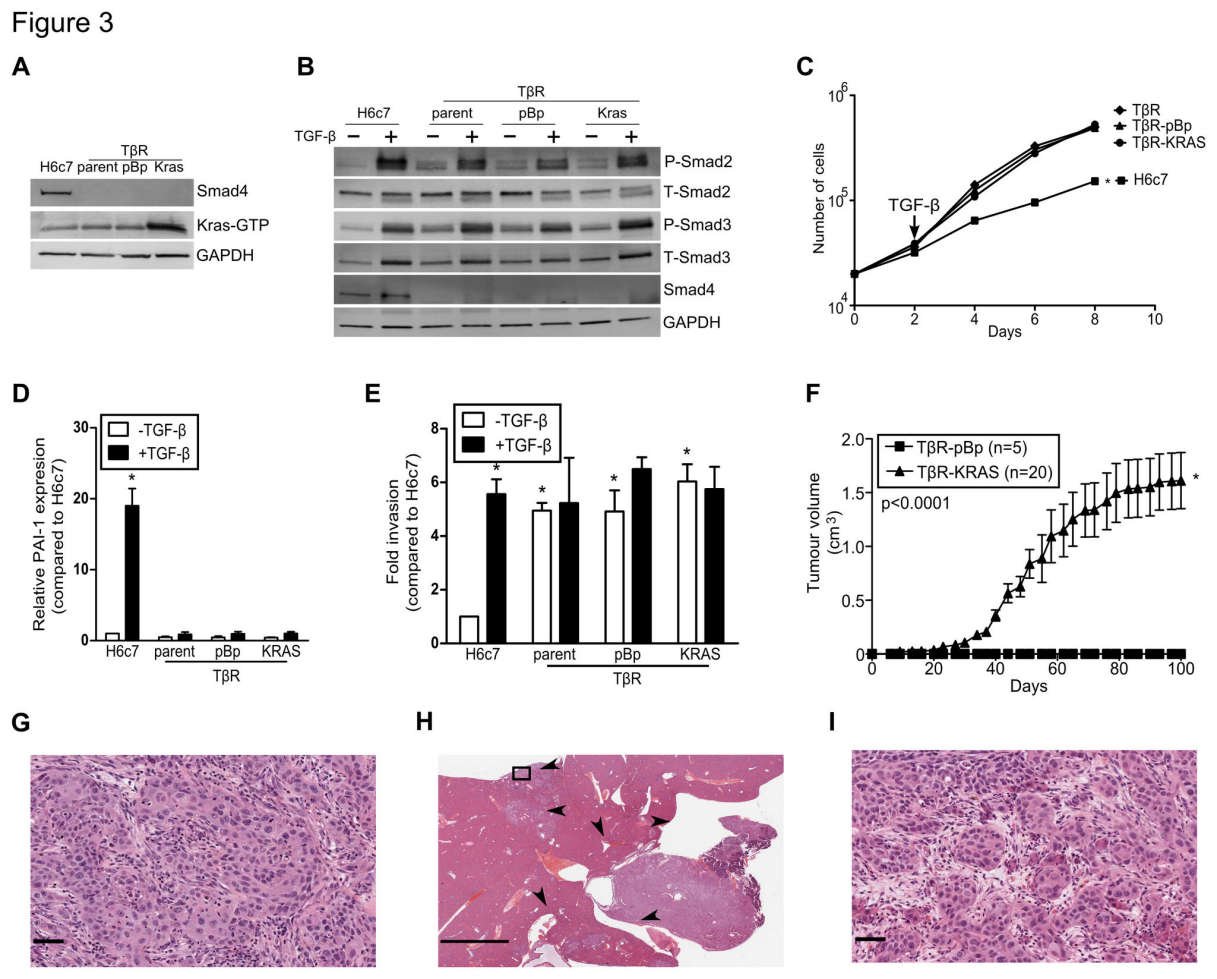
KRAS^G12V^-mediated transformation of the TβR cell *line*. (**A**) Immunoblots of activated RAS and Smad4. GAPDH was used as a loading control. (**B**) Immunoblots of phospho- and total Smad2/3, and Smad4. GAPDH was used as a loading control. (**C**) Growth curves of H6c7, TβR, TβR pBp, and TβR KRAS with TGF-β. (**D**) PAI-1 mRNA expression was assessed after 48 hours of TGF-β in the H6c7 and TβR cell lines (n=3). (**E**) Invasion assays through Matrigel coated membranes incubated with and without TGF-β (n=6). (**F**) Tumor growth curve after subcutaneous implantation of the TβR-pBp and TβR-KRAS cell lines into NOD-SCID mice (n=20). Representative H&E sections of orthotopic TβR-KRAS (**G**) xenografts and (**H** and **I**) metastases found in the liver and spleen. (* denotes significant differences between H6c7 and TβR cell lines or treated compared to vehicle where appropriate, one-way or two-way ANOVA, or linear regression where appropriate, p<0.05; data are presented as mean ± SEM).

Stable *KRAS* ^G12V^ expression in the TβR cell line was achieved using an amphotropic retrovirus (Figure 3A and Figure S2A). *KRAS* activity was markedly higher in the TβR-KRAS cell line, but this did not manifest in enhanced proliferation rate, as compared to control TβR-pBp line (Figure 3A and Figure S2B). Copy number analysis revealed a 45% decrease in the number of *Smad4* copies in the TβR-KRAS cell line and *Smad4* promoter methylation as revealed by MSP analysis demonstrated that *Smad4* gene expression was silenced by promoter methylation (Figure S2C). In contrast, *Smad2* mRNA expression remained unchanged. Decreases in *Smad3* mRNA expression were found in the TβR-pBp and TβR-KRAS lines, but this had no effect on protein expression (Figure 3B; and Figure S2D). *TGFBR2* expression was reduced by 45% in the TβR line and *KRAS* expression further decreased *TGFBR1* expression by 74%, but the decline in receptor expression had no effect receptor activated Smad phosphorylation (Figure 3B). The TβR-KRAS cell line maintained insensitivity to mito-inhibitory effects of TGF-β similarly to the parental TβR line (Figure 3C). Importantly and in contrast to the H6c7-S4KD2 cell line, *PAI-1* and *Smad7* were not induced after TGF-β treatment of the TβR line (Figure 3D and Figure S2E). The TβR cells constitutively showed a 5-fold higher invasive ability compared to the Hc67 cell line (n=6; p<0.05; Figure 3E). However, neither *KRAS* ^G12V^ expression nor TGF-β stimulation further increased invasiveness, suggesting that the TβR cell lines have reached maximal invasive capacity that is attainable by the H6c7 cells. 

Subcutaneous implantation of the TβR and TβR-pBp cell lines failed to form tumors in non-obese diabetic (NOD)-severe combined immune deficient (SCID) mice (n=5; Table 1). In contrast, the subcutaneous and orthotopic implantation of the TβR-KRAS cell line into the NOD-SCID mice led to tumor formation with complete penetrance (n=20; Figure 3F). Histology of the subcutaneous and orthotopic tumors formed by the TβR-KRAS cells displayed a poorly differentiated carcinoma (Figure 3G and Figure S2F). Importantly, metastases were identified in the liver and spleen of 15% and 77% of the mice, respectively (Figure 3H and Figure S2G). Examining the metastases revealed similar histology to the primary orthotopic tumor (Figure 3I and Figure S2H). 

### Smad4 restores TGF-β sensitivity in the TβR cell lines

**Figure 4:**
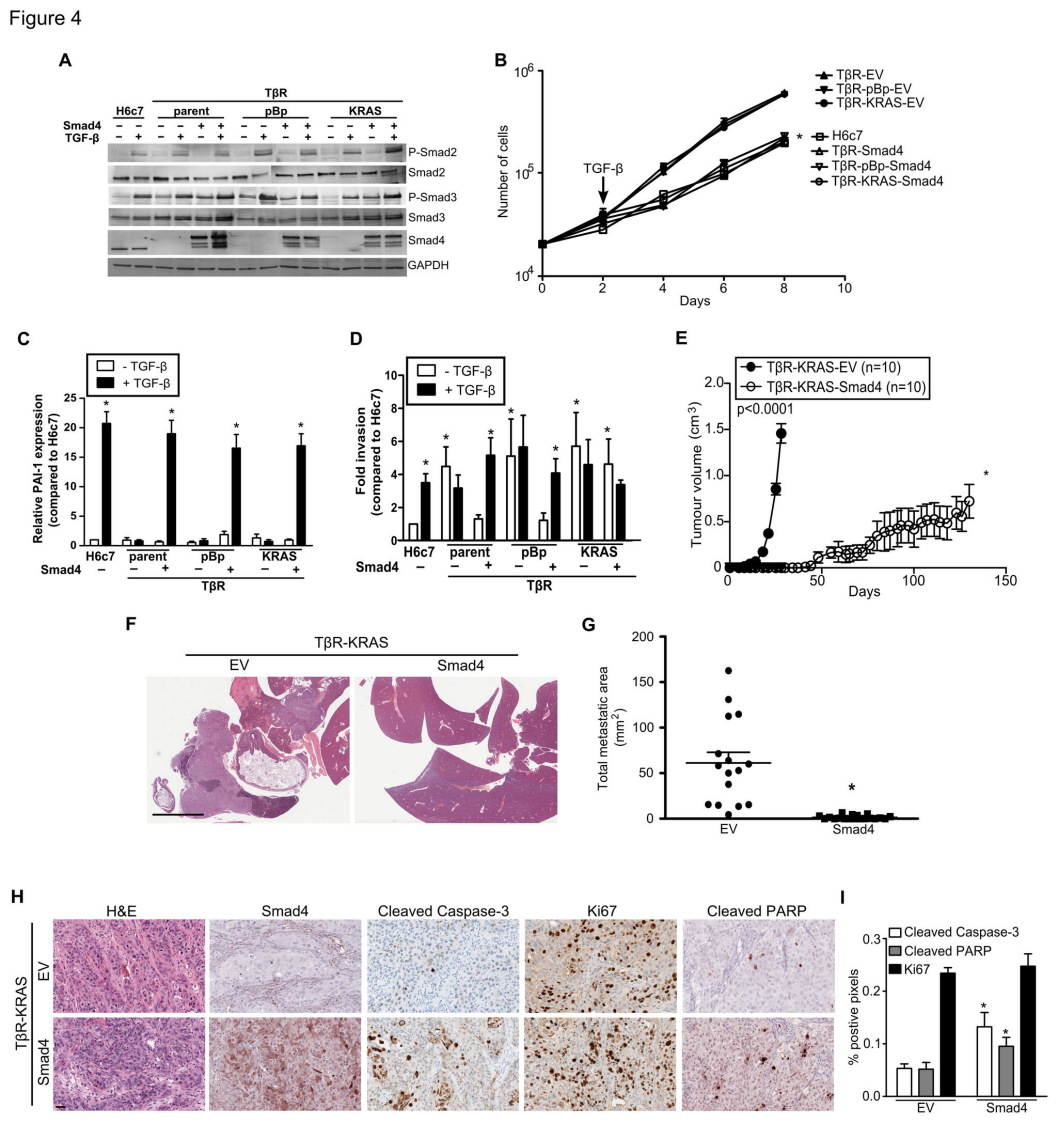
Smad4 expression restores TGF-β sensitivity and represses tumorigenicity in the TβR-KRAS cell *line*. (**A**) Immunoblots of phospho- and total Smad2/3, and Smad4. GAPDH was used as a loading control. (**B**) Growth curves of H6c7, TβR, TβR pBp, and TβR KRAS after restoration of Smad4 with TGF-β. (**C**) PAI-1 mRNA expression after 48 hours of TGF-β stimulation after forced Smad4 expression (n=3). (**D**) Invasion assays through Matrigel coated membranes incubated with and without TGF-β (n=6). (**E**) Tumor growth curves of TβR-KRAS- EV and TβR-KRAS-Smad4 (n=10). (**F**) The liver and spleens after orthotopic implantation of the TβR-KRAS and TβR-KRAS-Smad4 cell line. Scale bars represent 5 mm. (**G**) Total metastatic area of each TβR-KRAS-EV and TβR-KRAS-Smad4 orthotopic model. (**H**) Representative histological images of xenografts formed by TβR-KRAS-EV (n=16) and – Smad4 (n=19) cells after H&E, and immunostaining for Smad4, cleaved caspase-3, and Ki67. Scale bars represent 50 μm. (**I**) Quantification of Ki67, cleaved caspase-3, and cleaved PARP positive pixels of the TβR KRAS EV and – Smad4 xenografts. (* denotes significant differences between H6c7 and TβR cell lines or treated compared to vehicle where appropriate, two-way ANOVA, student t-test, or linear regression where appropriate, p<0.05; data are presented as mean ± SEM) .

*Smad4* has been previously demonstrated to be a potent tumor suppressor [20,21]. To determine if *Smad4* re-expression would suppress TβR-KRAS tumorigenicity, we stably expressed *Smad4* using a lentiviral construct fused with GFP (Figures 4A and Figure S3A). *Smad4* expression restored *TGFBR1* and *TGFBR2* mRNA expression in the TβR-KRAS cell line, and did not alter growth rate, but did sensitize the TβR cell lines to TGF-β (Figure 4B and Figure S3B). *Smad4* expression in the TβR lines reinstated TGF-β induced expression of *PAI-1* and *Smad7* (n=3; p>0.0001; Figure 4C and Figure S3C). Smad4 restoration attenuated the invasive phenotype of the TβR and TβR-pBp cell lines, but not in the TβR-KRAS line (n=6; Figure 4D), as *KRAS* ^G12V^ alone could increase H6c7 invasiveness. TGF-β treatment significantly promoted invasion in the TβR-Smad4 and TβR-pBp-Smad4 cell lines (p<0.05), but did not further enhance TβR-KRAS-Smad4 invasiveness. Although the TβR cell line displayed enhanced invasion through Matrigel coated Boyden chambers, the expression of E-cadherin and Snail2 was unchanged and that of vimentin was significantly decreased as compared to the H6c7 cell line (Figure S3D-F). In contrast, treatment of the H6c7 and Smad4 expressing TβR cell lines with TGF-β enhanced invasion, and this was associated with changes in the expression of E-cadherin, vimentin and Snail2 that are consistent with EMT.

### Smad4 re-expression causes marked inhibition in tumorigenicity, metastasis, and survival

Palpable masses were detected in NOD-SCID mice eight days after subcutaneous implantation of the TβR-KRAS-EV cell line (n=10; Figure 4E). In contrast, *Smad4* expression significantly delayed TβR-KRAS xenograft growth and palpable masses were first detected 41 days after implantation (n=10; p<0.0001; Figure 4E). Mice bearing TβR-KRAS-Smad4 xenografts had an increase in median survival from 27.5 days to 73 days compared to the TβR-KRAS-EV model (p<0.0001; Figure S3G). At the time of sacrifice, the mean weight of the TβR-KRAS-Smad4 xenografts were significantly lower than the TβR-KRAS-EV tumors (p<0.01; Figure S3H). 

Analogous findings of increased survival were found after orthotopic implantation of TβR-KRAS-Smad4 cells into NOD-SCID pancreases (n=19; p<0.001; Figure S3I). Mice bearing the TβR-KRAS-Smad4 xenografts had an increased median survival of 15 days in contrast to animals bearing the TβR-KRAS-EV xenografts (p<0.001). Mean tumor weight was significantly lower in the TβR-KRAS-Smad4 xenografts compared to the TβR-KRAS model (p=0.03; Figure S3J). Metastatic spread to the kidneys and spleen was significantly reduced in the orthotopic TβR-KRAS -Smad4 xenograft model (p<0.05; Table 1 and Figure 4F). Analyses of the orthotopic models revealed significantly reduced size and number of metastases per animal in the *Smad4* expressing model compared to the control TβR-KRAS-EV (p<0.01; Figure 4G and Figures S3K-L). 

 Immunoblotting and immunohistochemistry confirmed *Smad4* expression in the TβR-KRAS-Smad4 xenografts (Figure 4H and Figure S3M). No changes in Ki67 staining were detected; however *Smad4* expression in the xenografts was associated with increased cleaved caspase-3 and PARP (p<0.05; Figure 4I). These results suggest that *Smad4* expression delays tumor growth by promoting apoptosis. 

### Annotating the expression alterations in the TβR, TβR-KRAS, and TβR-KRAS- Smad4 reveal similar pathway changes in PDAC

**Figure 5:**
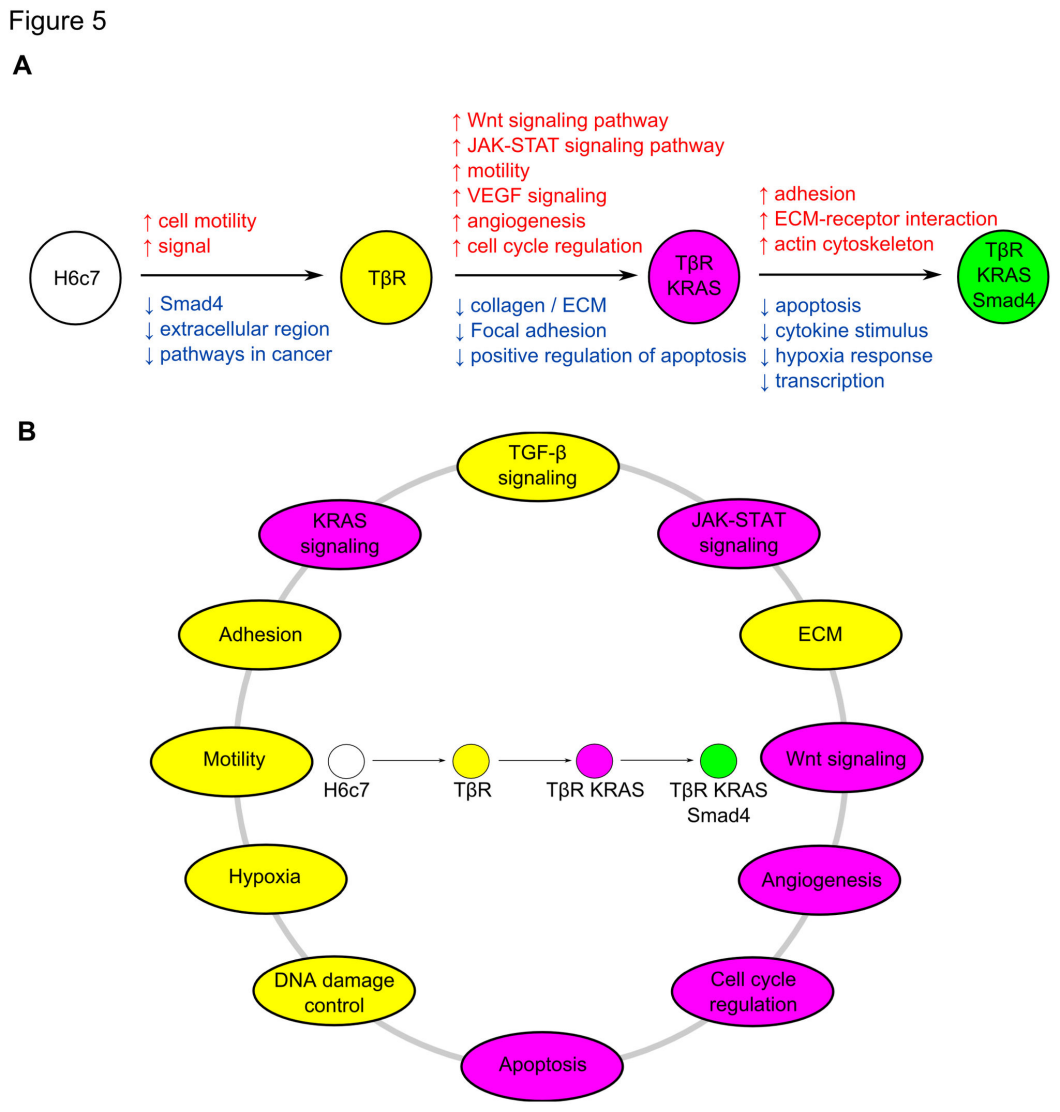
The gene expression changes due to Smad4 loss and KRAS^G12V^ expression. (**A**) The evolution from normal pancreatic duct epithelial cell to tumor cell line. The pathway alterations found in the analyses of the genomic and expression arrays between each transition are listed above the arrows. The red and blue alterations represent gains or upregulation, and losses or downregulation, respectively. (**B**) The pathways that were altered in the pancreatic duct cell carcinogenesis model. Yellow and purple corresponds to changes seen in the TβR and TβR-KRAS cell lines, respectively.

We examined the expression changes associated *Smad4* expression loss and with *KRAS* ^G12V^ expression. Clustering analysis of the top 400 differentially expressed genes revealed that introducing *KRAS* ^G12V^ in the TβR cell line caused greater gene expression alterations than acquiring TGF-β resistance in the H6c7 cell line (Figure S4A). The latter was associated with the upregulation of genes involved in cell motility, and downregulation of genes involved in extracellular region and pathways in cancer (Figures 5A, B; and Tables S2-S4 in File S1). *KRAS* ^G12V^ expression in the TβR cell line induced processes involved with Wnt and JAK/STAT signaling, angiogenesis, and motility, and downregulated genes involved in apoptosis, adhesion, and ECM. *Smad4* expression in the TβR-KRAS cell line enhanced gene expression in ECM-receptor interaction, ECM, and actin cytoskeleton regulation, and downregulation of hypoxia response and apoptosis.

## Discussion

The H6c7 cell line was originally established from normal human pancreatic duct epithelium [14,22]. In this study, we have demonstrated that *KRAS* ^G12V^ expression is insufficient for the full malignant transformation of H6c7 cells when residual *Smad4* signaling remains. In contrast, *KRAS* ^G12V^ can induce malignant transformation in a newly derived *Smad4* deficient H6c7-TβR cell line. As H6c7 cells have already manifested deregulated *Rb* and *p53* pathways, we conclude that Smad4 absence is obligatory and serves as a restriction point for *KRAS*-mediated malignant transformation of the HPDE cell line. We have also provided important evidence supporting the role of Smad4 loss in promoting metastasis in PDAC. 

The HPDE cell models are an important complement to pancreatic cancer GEMMs. These GEMMs that conditionally express *KRAS* ^G12D^ have established that oncogenic *KRAS* can initiate and promote pancreatic tumorigenesis in combination with other genetic aberrations [10,12,23,24]. While several *in vitro* studies have demonstrated that *KRAS* is necessary for maintenance of the neoplastic phenotypes in tumor cell lines, malignant transformation of normal pancreatic epithelial cells by oncogenic *KRAS* has proven to be more stochastic [25,26]. *KRAS* ^G12V^ can transform the human pancreatic Nestin positive epithelial (HPNE) cell line only after it had been immortalized by hTERT, HPV-E6E7, and small t antigen [27]. The incomplete transformation by *KRAS* ^G12V^ expression has also been reported in SV40 large T immortalized bovine pancreatic duct cells and primary rat pancreatic epithelial cells [28,29]. Our laboratory has previously reported that *KRAS* ^G12V^ expression in H6c7 cells using an ecotropic retroviral transduction system formed tumors with incomplete penetrance [13]. In this system, the prior introduction of the ecotropic receptor introduction led to tetraploidy development which permitted >10-fold *KRAS* ^G12V^ expression [13]. This is in contrast to our current amphotropic retroviral transduction system, which maintains paradiploidy after stable *KRAS* ^G12V^ expression, but limits *KRAS* expression to ~6-fold, and fails to tumor formation in SCID mice. 

Loss of *Smad4* expression itself has been found in approximately 50-55% of PDAC, most often by homozygous deletion or inactivating mutation [30,31]. However, a loss of heterozygosity involving chromosome 18q on which Smad4 gene is located has been found in a majority of PDAC [32]. Our finding that a complete abrogation of *Smad4* protein expression is essential in *KRAS/Smad4* driven malignant transformation of pancreatic duct epithelial cells suggests that more systematic and comprehensive analyses of *Smad4* inactivation from gene to protein level is warranted. The introduction of functional *Smad4* into the tumorigenic and metastatic TβR-KRAS line significantly suppresses tumorigenicity and metastasis, which emphasizes a strong tumor suppressive role in PDAC. The growth delay in *Smad4* expressing xenografts was associated with increased caspase-3 and PARP cleavage, which was independent of *p53* and *RB* since both proteins were inactivated in H6c7 cells after immortalization. Previous work using breast cancer cell line MDA-MB-468 has also reported that expression of *Smad4* can induce apoptosis in the absence of *p53* and *RB* [33]. Similarly, TGF-β sensitivity restoration by transfecting *TGFBR2* in PDAC cell line, MiaPaCA-2, upregulates expression of pro-apoptotic *Bax* [34]. These results suggest that one of the mechanisms by which Smad4 loss promotes pancreatic duct cell carcinogenesis is by promoting the anti-apoptotic pathway. 

Consistent with our own findings, *Smad4* has been shown to repress motility and invasion *in vitro* and its status has been associated with decreased metastasis in PDAC [8,21,35]. *In vitro*, reduction or absence of *Smad4* promotes invasion in the H6c7 and TβR cell lines, respectively. In agreement with our findings, decreasing *Smad4* expression increases invasiveness in the HPNE models [36]. The precise mechanism of how this phenotype is elicited requires further examination, and some of the proposed mechanisms have been attributed to regulation of *RON*, *EGFR*, and differential *Stat3* activation [36-38]. We did not observe alterations in *EGFR* and *RON* in our expression arrays, though gene expression analysis revealed enrichment in pathways involved in cell motility, cytoskeletal organization, axon guidance and ECM in the TβR cell line, which are congruent with the observed phenotypic differences in invasive ability. Loss of *Smad4* has recently been reported as presence of widespread metastasis in PDAC and is a poor prognostic marker in PDAC patients [8,39,40]. This is consistent with our data which demonstrates that *Smad4* expression suppresses metastasis in the orthotopic xenograft model. Collectively, these data demonstrate that *Smad4* loss drives invasion and metastasis in PDAC. Altogether, these findings are congruent to previous work examining the role of Smad4 in repressing tumor growth, metastasis, cellular invasion in established PDAC cell lines such as BxPC3, Hs766T, and Panc1 [20,21,41]. However, our study is the first to demonstrate that Smad4 loss is crucial in driving malignant transformation of normal pancreatic duct cells. 

Analysis of expression changes in the TβR/TβR-KRAS/TβR-KRAS-Smad4 cell lines identified the evolution of signaling pathway changes from normal duct to tumor cell that were also previously reported from the exomic sequencing of 24 invasive PDACs [42]. Alterations in the TGF-β, KRAS, JAK-STAT, and Wnt signaling pathways; cell adhesion; motility; ECM; cell cycle regulation; DNA damage; hypoxia, angiogenesis; and apoptosis were found after analyzing gene expression changes. Recently, 99 pancreatic tumors were sequenced and pathway analysis identified an enrichment of mutated genes in the axon guidance pathway [43]. Examination of the altered genes in the TβR and TβR-KRAS cell lines also revealed axon guidance genes, *SLIT2*, *SEMA3A*, and *EPHA*, thus indicating how this model of pancreatic duct cell carcinogenesis can recapitulate the types of pathway alterations seen in PDAC. These alterations have yielded promising insights into the requirements for tumorigenic transformation of the H6c7 cell line and further investigations into the identified genes may shed additional insight into the pathogenesis of this fatal cancer. Usage of the H6c7 cell line as a model of normal human pancreatic duct cells has provided a crucial platform to study the mechanistic roles of *KRAS* and *Smad4*. Our study clearly demonstrates that introducing the early genetic aberrations into normal pancreatic duct epithelial cells can recapitulate what has been observed PDAC, and is a key system for modeling molecular mechanism of human PDAC pathogenesis. 

## Supporting Information

Figure S1 **Stable Smad4 knockdown and KRAS^G12V^ expression.** (**A**) Smad4 mRNA was suppressed using four different shRNA constructs (KD1-4) and a nonsense (NS) in the H6c7 cell line. (**B**) Representative Western blots of Smad4 protein expression in Hc67 cells, where GAPDH is used as a loading control. (**C**) KRAS mRNA expression in H6c7 after introduction of NS and S4KD2. (**D**) Smad4 mRNA expression was suppressed after using S4KD2 shRNA construct in the H6c7 KRAS cell line. (**E**) Western blots of Smad4 and KRAS expression. (**F**) Smad and TGF-β receptors expression were assessed by qPCR and compared to the control H6c7 cell line (n=3). (**G**) Smad7 mRNA expression after 48 hours of TGF-β stimulation. Growth curves of (**H**) H6c7 NS and H6c7 S4KD2 (**I**) H6c7, H6c7 KRAS S4KDNS, and H6c7 KRAS S4KD2. Cells were treated with TGF-β on Day 2. (* denotes significant differences between the test and control samples student t-tests, and treated and untreated, one-way or two-way ANOVA and Bonferroni’s post hoc tests, and linear regression where appropriate, p<0.05, n=3.) .
(TIF)

Figure S2 **KRAS^G12V^ expression in the TβR cell line.** (**A**) KRAS mRNA expression in H6c7 and TβR cell lines (n=3). (**B**) Growth curves of H6c7, TβR, TβR pBp, and TβR KRAS. (**C**) Methylation specific PCR was performed on bisulfite treated gDNA isolated from H6c7 and TβR cells. Where U and M are denoted as unmethylated and methylated, respectively. (**D**) Smad and TGF-β receptors expression were assessed by qPCR and compared to the control H6c7 cell line (n=3). (**E**) Smad7 mRNA expression after 48 hours of TGF-β stimulation. Representative H&E section of a xenograft derived from (**F**) subcutaneous implantation and (**G** and **H**) orthotopic implantation demonstrating metastases found in the spleen as indicated by the arrowheads. Scale bars represent 50 μm and 5 mm, respectively. (* denotes significant differences between the test and control samples, treated and untreated samples; two-way ANOVA and linear regression where appropriate, p<0.05, n=3.) .
(TIF)

Figure S3 **Smad4 restoration in the TβR cell line.** (**A**) Smad and TGF-β receptors expression were assessed by qPCR and compared to the control H6c7 cell line (n=3).
(**B**) Growth curves of H6c7, TβR, TβR pBp, and TβR KRAS after restoration of Smad4. (**C**) Smad7, (**D**) E-Cadherin, (**E**) Snail2, and (**F**) Vimentin mRNA expression after 48 hours of TGF-β stimulation. Survival curves for the (**G**) subcutaneous and (**H**) orthotopic implantation of the TβR KRAS EV and TβR KRAS Smad4 in NOD SCID mice. Mean tumour volume for the (**I**) subcutaneous and (**J**) orthotopic xenograft models. Data is represented by mean ± SEM. Average (**K**) area and (**L**) number of metastases observed in the TβR KRAS EV and TβR KRAS Smad4 xenograft models. (**M**) Western blots of Smad4 and KRAS expression from the orthotopic xenograft samples. Data is represented by mean ± SEM. (* denotes significant differences between the test and control samples student t-tests, 2-way ANOVA, and linear regression where appropriate, p<0.05.) .
(TIF)

Figure S4 **Genomic and transcriptomic changes after acquiring TGF-β resistance and KRAS^G12V^ expression.** (**A**) Heatmap of hierarchical clustering used to analyse differential gene expression of the top 400 variable genes.
(TIF)

File S1 **Tables S1-S4.** Table S1. Primer sequences. Primer sequences for qPCR, MSP, Smad4 sequencing, and Smad4 copy number analysis. Table S2. Signaling pathways and processes that are altered during pancreatic duct cell carcinogenesis. Table S3. Analysis of upregulated genes compared to the H6c7 cell line. Changes in gene expression in the TβR cell lines were compared to the H6c7 cell line. Alterations in gene expression were categorised based on more than two-fold expression changes and examined using pathway and gene ontology classifications. Table S4. Analysis of downregulated genes compared n in the H6c7 cell line. Changes in gene expression in the TβR cell lines were compared to the H6c7 cell line. Alterations in gene expression were categorised based on more than two-fold expression changes and examined using pathway and gene ontology classifications.
(DOC)
