# Supplementary figures and images for: Loss of Canonical Smad4 Signaling Promotes KRAS Driven Malignant Transformation of Human Pancreatic Duct Epithelial Cells and Metastasis
Source: PLoS One. 2013 Dec 27;8(12):e84366. doi: 10.1371/journal.pone.0084366 (PMC3873993; doi:10.1371/journal.pone.0084366)

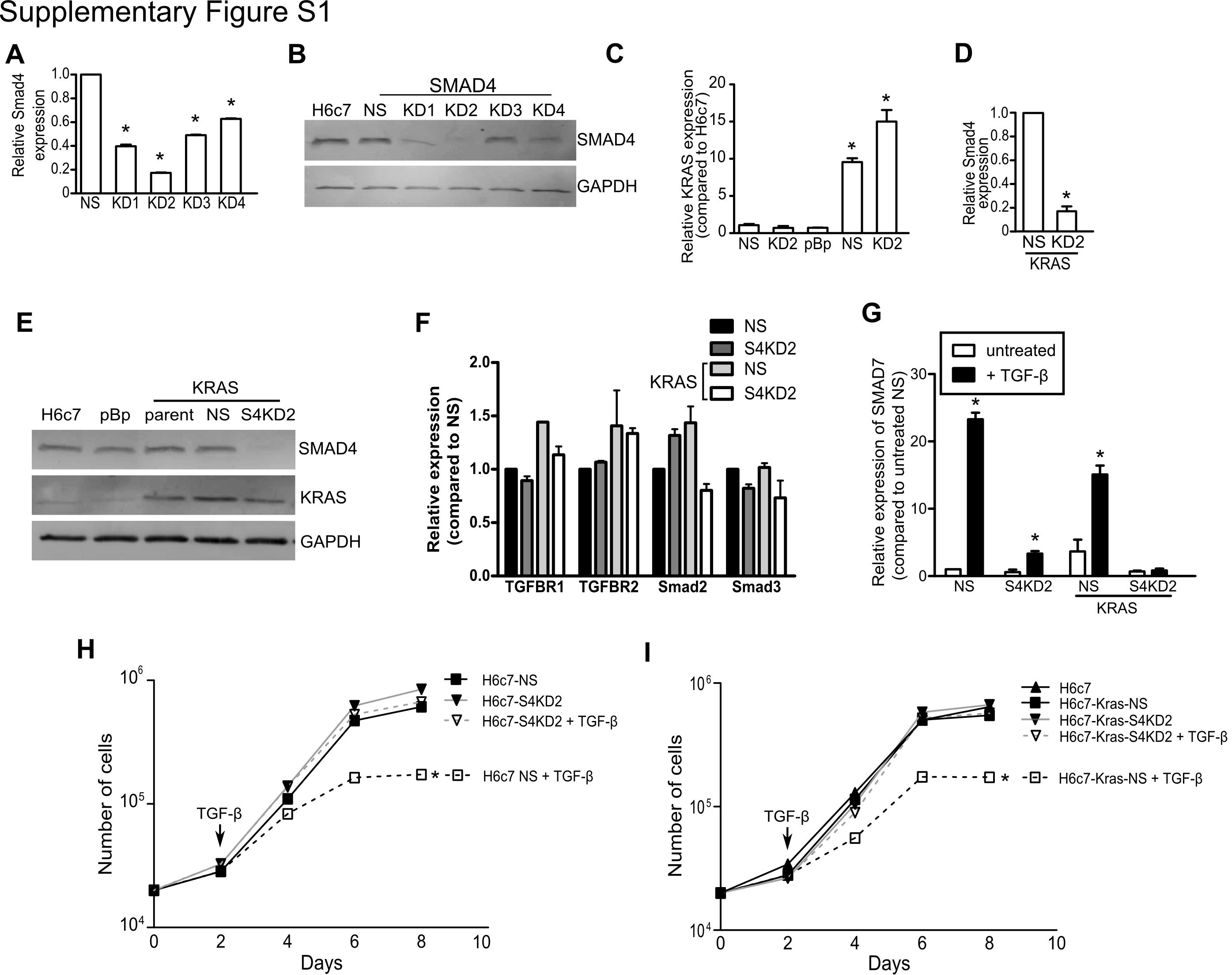

Supplement: Figure S1 — Stable Smad4 knockdown and KRASG12V expression. (A) Smad4 mRNA was suppressed using four different shRNA constructs (KD1-4) and a nonsense (NS) in the H6c7 cell line. (B) Representative Western blots of Smad4 protein expression in Hc67 cells, where GAPDH is used as a loading control. (C) KRAS mRNA expression in H6c7 after introduction of NS and S4KD2. (D) Smad4 mRNA expression was suppressed after using S4KD2 shRNA construct in the H6c7 KRAS cell line. (E) Western blots of Smad4 and KRAS expression. (F) Smad and TGF-β receptors expression were assessed by qPCR and compared to the control H6c7 cell line (n=3). (G) Smad7 mRNA expression after 48 hours of TGF-β stimulation. Growth curves of (H) H6c7 NS and H6c7 S4KD2 (I) H6c7, H6c7 KRAS S4KDNS, and H6c7 KRAS S4KD2. Cells were treated with TGF-β on Day 2. (* denotes significant differences between the test and control samples student t-tests, and treated and untreated, one-way or two-way ANOVA and Bonferroni’s post hoc tests, and linear regression where appropriate, p<0.05, n=3.) . (TIF) [file pone.0084366.s001.tif]

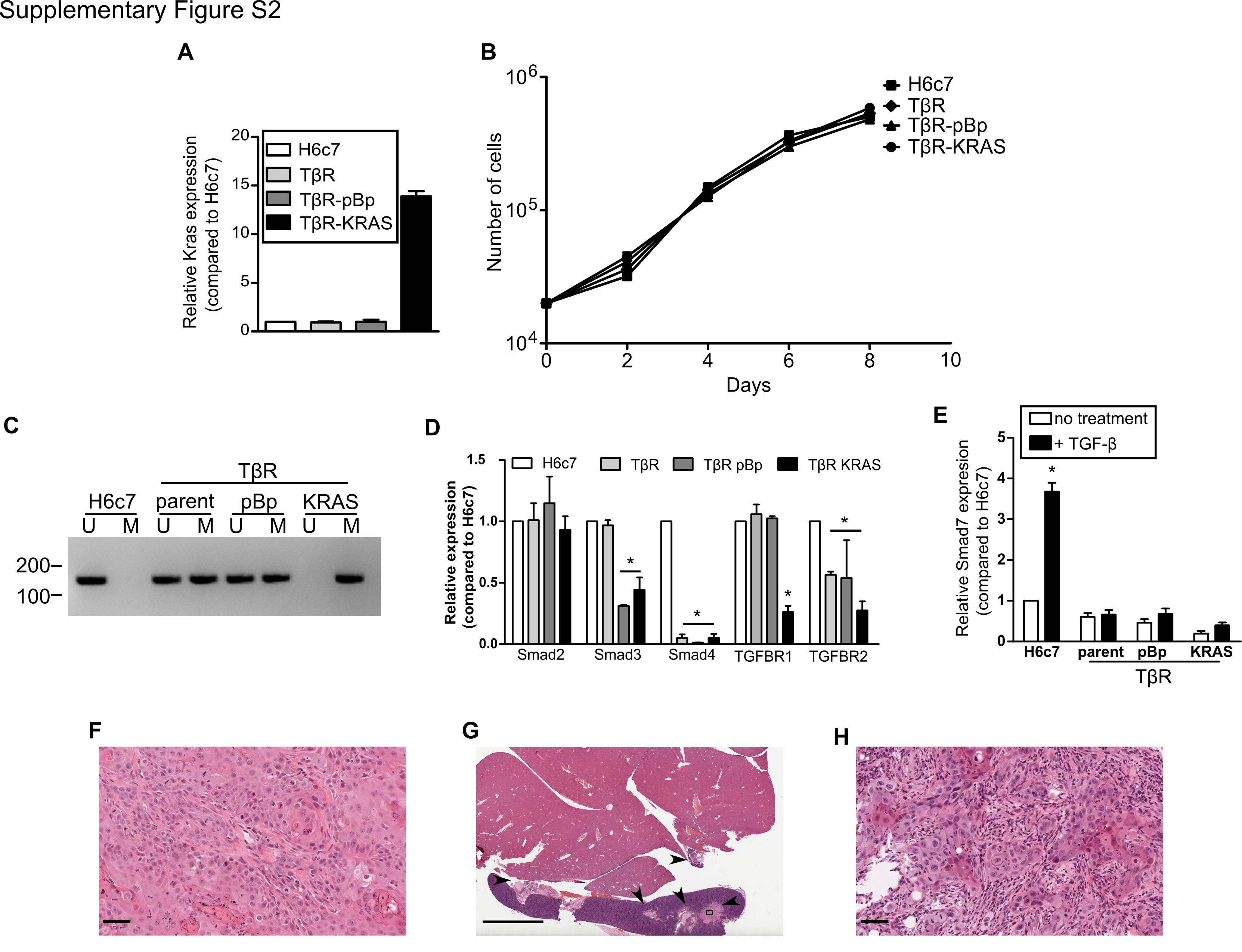

Supplement: Figure S2 — KRASG12V expression in the TβR cell line. (A) KRAS mRNA expression in H6c7 and TβR cell lines (n=3). (B) Growth curves of H6c7, TβR, TβR pBp, and TβR KRAS. (C) Methylation specific PCR was performed on bisulfite treated gDNA isolated from H6c7 and TβR cells. Where U and M are denoted as unmethylated and methylated, respectively. (D) Smad and TGF-β receptors expression were assessed by qPCR and compared to the control H6c7 cell line (n=3). (E) Smad7 mRNA expression after 48 hours of TGF-β stimulation. Representative H&E section of a xenograft derived from (F) subcutaneous implantation and (G and H) orthotopic implantation demonstrating metastases found in the spleen as indicated by the arrowheads. Scale bars represent 50 μm and 5 mm, respectively. (* denotes significant differences between the test and control samples, treated and untreated samples; two-way ANOVA and linear regression where appropriate, p<0.05, n=3.) . (TIF) [file pone.0084366.s002.tif]

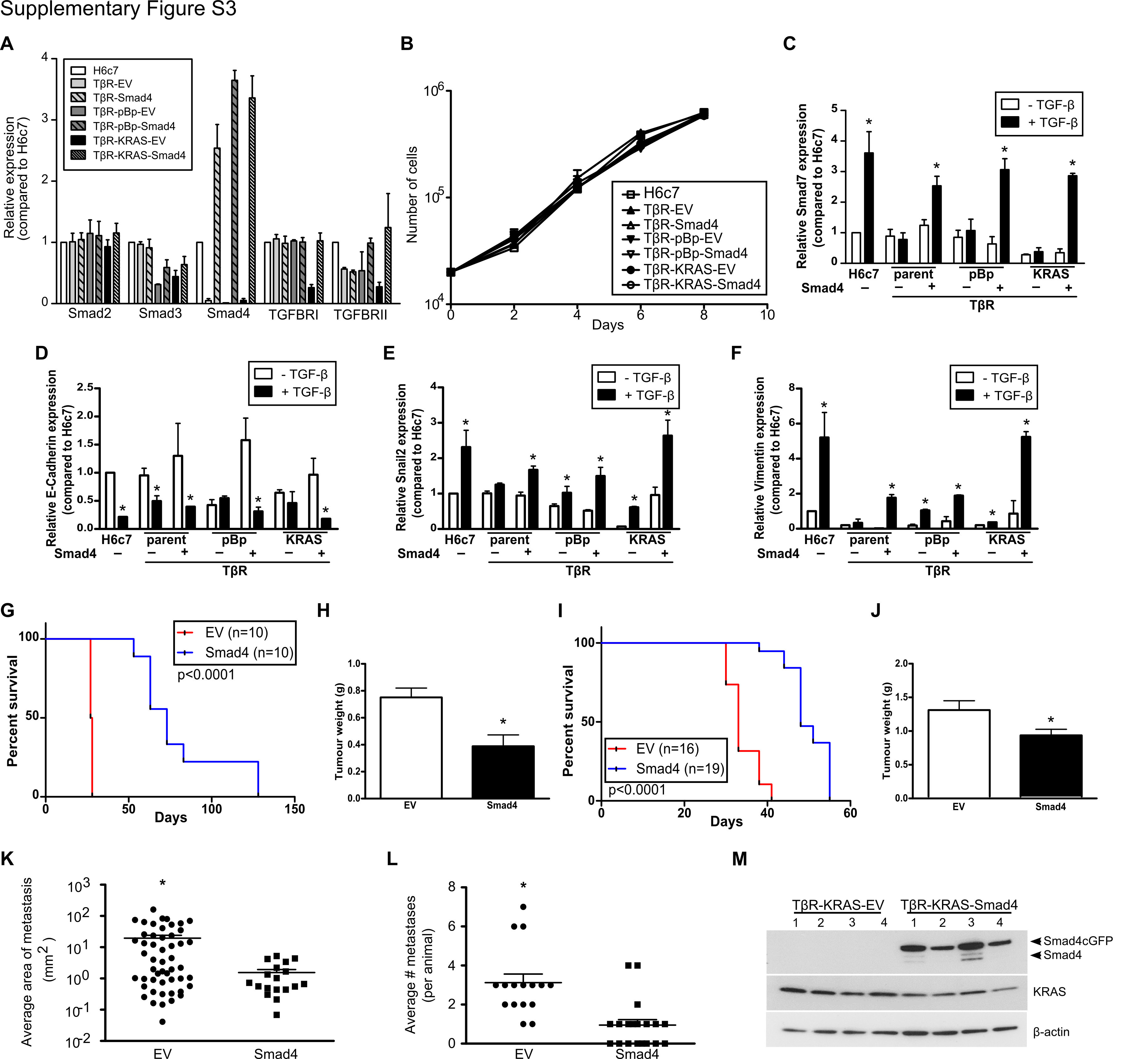

Supplement: Figure S3 — Smad4 restoration in the TβR cell line. (A) Smad and TGF-β receptors expression were assessed by qPCR and compared to the control H6c7 cell line (n=3). (B) Growth curves of H6c7, TβR, TβR pBp, and TβR KRAS after restoration of Smad4. (C) Smad7, (D) E-Cadherin, (E) Snail2, and (F) Vimentin mRNA expression after 48 hours of TGF-β stimulation. Survival curves for the (G) subcutaneous and (H) orthotopic implantation of the TβR KRAS EV and TβR KRAS Smad4 in NOD SCID mice. Mean tumour volume for the (I) subcutaneous and (J) orthotopic xenograft models. Data is represented by mean ± SEM. Average (K) area and (L) number of metastases observed in the TβR KRAS EV and TβR KRAS Smad4 xenograft models. (M) Western blots of Smad4 and KRAS expression from the orthotopic xenograft samples. Data is represented by mean ± SEM. (* denotes significant differences between the test and control samples student t-tests, 2-way ANOVA, and linear regression where appropriate, p<0.05.) . (TIF) [file pone.0084366.s003.tif]

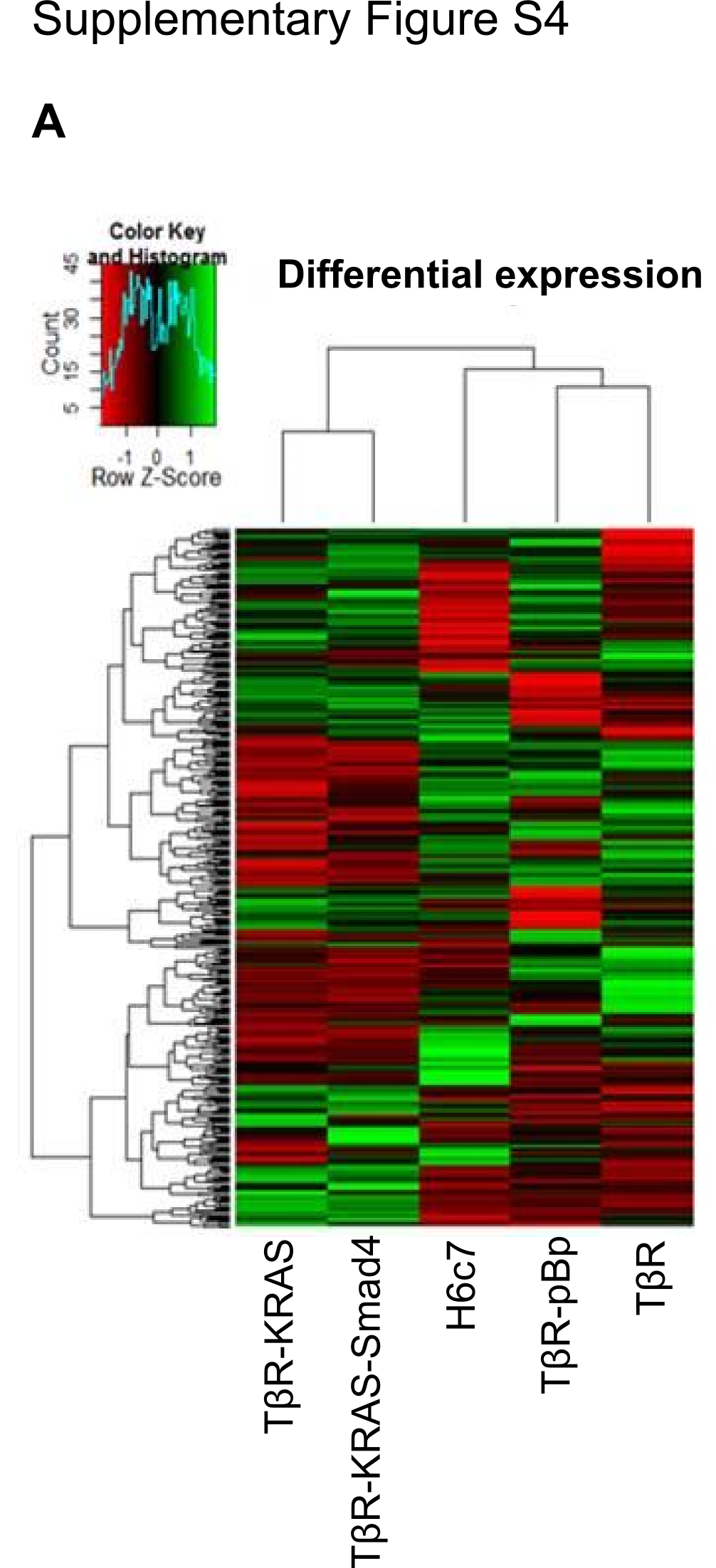

Supplement: Figure S4 — Genomic and transcriptomic changes after acquiring TGF-β resistance and KRASG12V expression. (A) Heatmap of hierarchical clustering used to analyse differential gene expression of the top 400 variable genes. (TIF) [file pone.0084366.s004.tif]
