# Supplementary material for: Loss of Canonical Smad4 Signaling Promotes KRAS Driven Malignant Transformation of Human Pancreatic Duct Epithelial Cells and Metastasis
Source: PLoS One. 2013 Dec 27;8(12):e84366. doi: 10.1371/journal.pone.0084366 (PMC3873993; doi:10.1371/journal.pone.0084366)
Supplement: File S1 — Tables S1-S4. Table S1. Primer sequences. Primer sequences for qPCR, MSP, Smad4 sequencing, and Smad4 copy number analysis. Table S2. Signaling pathways and processes that are altered during pancreatic duct cell carcinogenesis. Table S3. Analysis of upregulated genes compared to the H6c7 cell line. Changes in gene expression in the TβR cell lines were compared to the H6c7 cell line. Alterations in gene expression were categorised based on more than two-fold expression changes and examined using pathway and gene ontology classifications. Table S4. Analysis of downregulated genes compared n in the H6c7 cell line. Changes in gene expression in the TβR cell lines were compared to the H6c7 cell line. Alterations in gene expression were categorised based on more than two-fold expression changes and examined using pathway and gene ontology classifications. (DOC) [file pone.0084366.s005.doc]

Table S1 – Primer Sequences

| Assay | Gene Name / exon | Forward | Reverse |
| --- | --- | --- | --- |
| qPCR | RPS13 | gttgctgttctaaagcatcttg | aatatcgagccaaacggtgaa |
|  | TBP | gggcattatttgtgcactgaga | tagcagcacggtatgagcaact |
|  | ACTB | tcctaaaagcaccccacttct | gggagaggactgggccatt |
|  | Smad4 | cctgttcacaatgagcttgcat | cctacctgaacatccatttcaa |
|  | KRAS | caggctcaggacttagcaagaag | tgttttcgaatttctcgaactaatgta |
|  | Smad7 | cccgatggattttctcaaacc | ccagataattcgttcccctg) |
|  | PAI-1 | cagctcatcagccactggaaa | atgtcggtcattcccaggttc |
|  | Smad2 | atctttgtcgacagccccaa | gccagaagagcagcaaattcc |
|  | Smad3 | tctccaatgtcaacaggatgc | aagacctcccctccgatgtagt |
|  | TGFBR1 | ctgcaatcaggaccattgca | accaaggccaggtgatgactt |
|  | TGFBR2 | aacggacaggaagtctgtgtgg | tgcactttggagaagcagcat |
| qPCR – Copy Number Analysis | Smad4 Primer Set 1 | tgtgcccatagacaaggtgga | catgaggaaatcctttccga |
|  | Smad4 Primer Set 2 | gattgcagacccacaacctt | agccatgcctgacaagttct |
|  | Smad4 Primer Set 3 | tgtgtgacaccaccctccta | gagctctgaatggatccagcc |
| Methylation Specific PCR | Methylated | ttataggcgtgtgttattatgatcg | catcactttaaaaaaccaaaacga |
|  | Unmethylated | tatacctgtgtgtttattatgattgg | catcactttaaaaaaccaaaacaaaa |

Table S2: Signaling pathways and processes altered during pancreatic duct cell carcinogenesis

| Process | Number of genes | Representative genes |
| --- | --- | --- |
| TGF-β signaling | 4 | SMAD4, ID2, TGIF1, TGFBR2 |
| JAK-STAT signaling | 7 | LIF, CSF3, CBLC, SPRY1, IL29, IL4R, SPRY4 |
| ECM | 40 | COL4A4, FGF1, FGF5, FN1, ITGA1, ITGB6, ITGB8, LAMA1, MMP9, MMP28, SERPINB2 |
| Wnt signaling | 7 | FZD8, WNT10B, DKK1, CXXC4, CAMK2B, FOSL1, WNT7A |
| Angiogenesis | 17 | ANGPTL4, FGF5, THBS1, VEGFC |
| Cell cycle regulation | 5 | CDKN1C, CDKN2C, KAT2B, PML |
| Apoptosis | 15 | BCL-2, CASP14, DAPK2, FOXO3, MCL-1 |
| DNA damage control | 4 | XRCC3, RAD51L1, BRCA2, RAD54L |
| Hypoxia | 4 | KCNMA1, SOCS3, EDN1, NR4A2 |
| Motility | 30 | FYN, IL6, RAC2, S100A9, S100P, SLIT2, SMO, VEGFC |
| Adhesion | 20 | CCDC80, CD274, CLDN8, CLDN16, COL1A1, COL4A4, COL5A3, COL8A1, CX3CL1, DLL1, FN1, LAMA1, ITGB8, KDR, |
| KRAS signaling | 12 | AKT3, DUPS2, DUSP4, DUSP6, FOS, KRAS, RAC2 |

Table S3. Analysis of upregulated genes compared to the H6c7 cell line

| Sample | Process | Genes |
| --- | --- | --- |
| TβR | extracellular region | VIP, CTHRC1, DCD, CCK, CRELD2, EPDR1, TIMP4, CX3CL1, GPC4, CALCB, RSPO4, COL9A2, ST3GAL2, SERPINA1, SPATA6, MUC15, EBI3, MUC1, IL6, LCN12, KRTDAP, IGF2, TCN1, NPTXR, INHBE, MFAP2, MFAP4 |
|  | cell motility | CTHRC1, SMO, IL6, CCK, S100P, FYN, PAX6, NEUROG2, DNAH1, CX3CL1 |
|  | positive regulation of cell differentiation | SMO, IL6, FOXA2, HOXA11, SOX2, PPARG, MAP1B, PAX6, EOMES, IGF2 |
| TβR KRAS | Wnt signaling pathway | FZD8, WNT10B, DKK1, CXXC4, CAMK2B, FOSL1, WNT7A, |
|  | Jak-STAT signaling pathway | LIF, CSF3, CBLC, SPRY1, IL29, IL4R, SPRY4 |
|  | Cell motion | PLAT, S100P, NDN, PODXL, S100A9, CCL5, CDH4, PLAUR, VEGFC, EPHA4, ID1, FOXG1, HBEGF, SEMA3B, POU4F1, SEMA3A, THBS1, FGF2, ENG, ETV4, CEACAM1, NR2F1, ARHGDIB |
|  | angiogenesis | PLAT, CDX2, WT1, EDNRA, VEGFC, SH2D2A, ID1, THBS1, TNFAIP2, ENG, FGF2, CEACAM1, ANGPTL4, PTGS2, NOS3, AKT3, PIK3R1 |
|  | axonogenesis | EPHA4, SLITRK4, NDN, FOXG1, UCHL1, SEMA3B, POU4F1, SEMA3A, CDH4, ETV4 |
|  | MAPK signaling | FOS, DUSP4, RPS6KA6, BDNF, ATF4, CACNA2D1, DUSP2, RAC2, NR4A1, FGF12, DUSP6 |
| TβR KRAS Smad4 | Cell adhesion molecules (CAMs) | NRXN2, IL32, L1CAM, ITGB2, PCDHB12, MCAM, PCDH18, AMIGO2, CD36, ITGB6, MSLN, VCAN, MFAP4, FN1 |
|  | ECM-receptor interaction | CD36, ITGB6, SV2A, FN1, MMP9, FST, IL32, C1R, C1S, CXCL10, SPINK7, FLT3LG, SAA2, SAA1, SBSN, MSLN, SERPINA3, CDA, SCG5, PNLIPRP3, FN1, MATN2, MATN3, CFB, EFEMP2, KRTDAP, CCDC80, C10ORF99, DLL1, A2ML1, PROC, CXCL14, CST6, SERPINF2, TFPI, IGFL1, MFAP2, VCAN, MFAP4 |
|  | Regulation of actin cytoskeleton | FGFR1, ITGB6, ITGB2, MYH14, FGD3, FN1 |
|  | Response to wounding | S100A8, CFB, EFEMP2, ITGB2, C1R, GPR68, C1S, PROC, S100A12, CXCL10, ALOX15, CD36, SAA2, SAA1, SERPINF2, ITGB6, SERPINA3, TFPI, VCAN, FN1 |

Table S4. Analysis of downregulated genes compared to the H6c7 cell line

| Sample | Process | Genes |
| --- | --- | --- |
| TβR | extracellular region | FGF5, MMP28, CCL5, CXCL10, NETO1, PCSK1, WNT4, SAA2, SAA1, SFTPD, SPON2, FGF1, DEFB1, PNLIPRP3, ANGPTL4, COL4A4, FLRT2, ICAM4, CHI3L1, CILP, C10ORF99, NID1, SLIT2, CNGA1, VEGFC, DKK1, CXCL14, CST6, SERPINB2, CA2, IGFBP5 |
|  | cell motility | VEGFC, SAA2, NDN, ID1, SAA1, SFTPD, ISL1, CCL5, SLIT2, CXCL14, DEFB1, GNG7, CXCL10 |
|  | Pathways in cancer | COL4A4, VEGFC, FGF5, WNT4, TCEB2, SMAD4, FGF1 |
|  | TGF-β signaling pathway | SMAD4, ID2, TGIF1, TGFBR2 |
| TβR KRAS | ECM-receptor interaction | COL4A4, FLRT3, HAPLN3, ELN, CRTAP, CCDC80, SPOCK1, NID1, COL5A3, EMILIN2, SMOC2, LAMA1, WNT4, SFTPD, COL12A1, MFAP2, COL1A1, MFAP4, MFAP5, FN1 |
|  | Focal adhesion | SPOCK1, PCDHB12, SDC3, ITGB8, MSLN, COL12A1, GPNMB, FN1, PCDHGA12, FLRT3, SVEP1, HAPLN3, PTPRM, NRXN2, PDPN, NID1, COL5A3, EMILIN2, SIRPA, PCDH18, LAMA1, CD36, CDON, DSG1, ROR2, MFAP4, CDH10 |
|  | positive regulation of apoptosis | VAV3, HOXA13, PML, FOXO3, ZBTB16, DAPK2, ITSN1, PLAGL1, PRUNE2, APOE, CDKN2C, CASP14, SST, IGFBP3 |
|  | cell cycle arrest | PLAGL1, CDKN1C, KAT2B, CDKN2C, PML |
|  | DNA damage control | XRCC3, RAD51L1, BRCA2, RAD54L |
| TβR KRAS Smad4 | MAPK signaling pathway | FOS, DUSP4, DUSP2, NR4A1, FGF12 |
|  | Pathways in cancer | FOS, PTGS2, IL8, CYCS, FGF12 |
|  | Regulation of apoptosis | SERINC3, KCNMA1, BCL-2, RTN4, ING3, PTGS2, MCL1, SOCS3, CYCS, CIDEB, NR4A2, NR4A1, IGF2 |
|  | Response to hypoxia | KCNMA1, SOCS3, EDN1, NR4A2 |
